# Seroepidemiology of Measles, Mumps, Rubella and Varicella in Italian Female School Workers: A Cross-Sectional Study

**DOI:** 10.3390/vaccines9101191

**Published:** 2021-10-16

**Authors:** Nicola Frau, Federico Meloni, Jacopo Fostinelli, Laura Portas, Igor Portoghese, Emma Sala, Ilaria Pilia, Luigi Isaia Lecca, Giuseppe De Palma, Marcello Campagna

**Affiliations:** 1 Department of Medical Sciences and Public Health, University of Cagliari, Cittadella Universitaria di Monserrato, SS 554 bivio per Sestu, Monserrato, 09042 Cagliari, Italy; n.frau4@studenti.unica.it (N.F.); igor.portoghese@unica.it (I.P.); ilaria.pilia@ca.omceo.it (I.P.); luigiisaia.lecca@unifi.it (L.I.L.); mcampagna@unica.it (M.C.); 2 Department of Medical and Surgical Specialties, Radiological Sciences and Public Health, University of Brescia, 25123 Brescia, Italy; jacopo.fostinelli@unibs.it (J.F.); emma.sala@unibs.it (E.S.); giuseppe.depalma@unibs.it (G.D.P.); 3 National Heart and Lung Institute, Imperial College London, London SW7 2AZ, UK; l.portas18@imperial.ac.uk

**Keywords:** early childhood teacher, school teacher, protective immunity, vaccine-preventable disease, public health surveillance, seroprevalence

## Abstract

Background: Determining the proportion of susceptible workers can represent a first step to the biological risk assessment related to measles, mumps, rubella and varicella exposure. This study aimed to assess the immunity against measles, mumps, rubella and varicella viruses in a cohort of female school workers. Methods: A cross-sectional seroepidemiological study in a sample of 263 school workers undergoing routine annual workplace health surveillance program was conducted. As part of the health surveillance program, serum samples were collected and tested for measles, mumps, rubella and varicella IgG antibodies. Results: Overall seropositivity was 90.5%, 85.2%, 94.7% and 97.3% for measles, mumps, rubella and varicella, respectively. In relation to mumps occupation-specific seropositivity, a statistically significant difference was observed, showing the lowest prevalence of protected individuals in other occupation groups. Moreover, in relation to rubella, school workers born in Centre Italy had the lowest seropositivity of protective antibodies and the difference between groups was statistically significant. Measles and rubella seropositivity showed a significant decrease after 2015. Conclusions: This study showed a relevant proportion of school workers susceptible to the aforementioned diseases. These results highlighted the need for proper health surveillance and immunological controls in school workers, especially for females, and provided useful insights to policymakers to select effective strategies aimed at containing the risk of vaccine-preventable diseases at schools.

## 1. Introduction

Despite the efforts of the World Health Organization (WHO) programs to eliminate vaccine-preventable diseases worldwide, measles, mumps, rubella and varicella are still not eradicated and have even re-emerged with recurrent outbreaks both in developed and industrialized countries [1].

Vaccination rates have fallen to dangerously low levels in certain communities due to the effects of a widespread vaccine hesitancy that, as of today, remains not restricted to any specific region or continent but exists worldwide [2,3]. Despite the WHO European Region making substantial progress towards measles and rubella elimination over the past 5 years [4], in the period ranging from 2010 to 2016 the Italian childhood immunization rates steadily declined for both mandatory and recommended vaccines [5], leading to a decrease in herd immunity and to re-emerging outbreaks of vaccine-preventable diseases [6].

From January to August 2017 more than 4400 measles cases have been reported in Italy. The highest incidence was reported in infants below one year of age and 7% of cases occurred among healthcare workers (HCW). Three deaths occurred and two cases of encephalitis have been reported [7]. In the same year, from January to December, were observed 65 rubella cases, more than twice the cases reported during 2016 [8]; two of them were congenital and one in a pregnant woman [9]. Concerning mumps, the European Centre for Disease Prevention and Control (ECDC) reported 829 cases in Italy in 2017 [10]. The last Italian report on varicella epidemiological trend described 59,388 cases in 2013 [11].

In response to these dramatic concerns, the Italian Parliament approved in July 2017 law n.119 that established 10 vaccinations (anti-measles, anti-mumps, anti-rubella and anti-varicella included) as mandatory and free for children aged from 0 to 16. Because of this law, children had to be vaccinated for admission to childcare up to primary school, in order to contain the spread of vaccine-preventable diseases in school and protect both immunocompromised subjects and the population, thanks to the increase in herd immunity rates [12]. After the law approval, Signorelli et al. reported a significant increase in childhood vaccine coverage: in 1 year, measles vaccine coverage rates (used as a proxy for measles, mumps and rubella) raised from 87.3% to 91.7% [5].

Nonetheless, immunity rate gaps remain in Italian territory. In fact, in July 2019 the Italian Ministry of Health reported from January to July 2019 overall 1334 cases of measles with a median age of 30 years and a complication rate of 31%. The occupational sectors indicated as at higher risk were the healthcare and the school sector, with an increasing number of cases, occurred in HCW and school workers [6]. However, despite primary school and early childhood teachers being described as at higher risk to contract both contact and airborne infectious diseases [13], vaccination requirements have been approved only for students and not for the workers employed in the school sector. School environments are characterized by crowded classrooms, sometimes with air exchange provided by mechanical ventilation systems. In relation to those conditions, Kutter et al. reported that most secondary cases never came in direct contact with the index patient and some were never even simultaneously present in the same area as the index case. They also reported that measles virus can accumulate in environments (such as classrooms and hallways) and can be dispersed through the ventilation system [14]. In addition, school workers have frequent and prolonged close contacts with their pupils, especially in early childhood settings, where they are responsible for children hygiene [15]. In this regard, vaccine-preventable diseases can represent an occupational hazard for school workers, as they are for HCW [16,17]. Primary school and early childhood teachers can acquire infectious diseases from pupils or serve as a reservoir of infections for them. Moreover, once the disease has been contracted by the worker, it can be spread through his own susceptible familiars’ [18].

In this scenario, determining the proportion of school workers, especially teachers, susceptible to vaccine-preventable diseases can represent a first step to the biological risk assessment related to measles, mumps, rubella and varicella exposure and can provide useful data for policymakers, to establish the need for specific preventive measures, such as mandatory vaccinations, for this large group of workers.

This study aimed to assess the immunity against measles, mumps, rubella and varicella viruses in a cohort of Italian female school workers.

## 2. Materials and Methods

### 2.1. Study Design

A cross-sectional seroepidemiological study in a sample of school workers was conducted. For each worker the following information was extracted from the periodical health surveillance records: gender, date of birth, birthplace, job, measles, mumps, rubella and varicella IgG antibodies titers and date of antibody determination. Data were collected using a standardized data collection form.

### 2.2. Study Population

Workers were employed at scholar services, ranging from early childhood center (3 months to 3 years) to pre-primary school (from 3 to 5 years) and already under the periodical health surveillance as workers according to the Italian law D.Lgs. 81/08. Eligibility criteria were: to have been subjected to a medical examination and the health surveillance program as a school worker in the period 2001–2019, and availability of serological testing for measles, mumps, rubella and varicella. The study population was assorted by place of birth (North Italy, Centre Italy, South Italy and major Islands, and Foreign Country), occupation (teacher, early childhood educator and other occupation, which mostly included janitors and cafeteria workers), and age at the date of the antibody determination (under 30, from 30 to 39, from 40 to 49, and equal or more than 50, according to Koivisto et al.) [19]. As the year 2015 was previously set as a target date for the elimination of measles and rubella in the WHO European Region by World Health Organization [20], subjects were also categorized into two groups based on the date of the antibody determination (before and after 2015). The choice was made to assess whether, after the target date, the rate of protected individuals was increased. Birthplace and age groups were adapted to assess the multi-variate logistic regression analysis. Because of the expected low number of workers in birthplace groups and in order to have greater balance among group dimensions, we incorporate Centre Italy and South Italy and Islands groups into the Rest of Italy group. Foreign Country group was not considered computing logistic regression. The decision of excluding foreign country group was taken due to the fact that birthplace groups were considered mainly to assess the heterogeneous vaccination programs along the Italian territory. Concerning age groups, under 30, from 30 to 39, from 40 to 49, and equal or more than 50 groups were incorporated, resulting in new <40 and ≥40 groups.

### 2.3. Laboratory Methods

As part of the health surveillance program, serum samples were collected and tested for measles, mumps, rubella and varicella IgG antibodies. Serological analyses were performed in the Laboratory of Microbiology of the Hospital of Brescia “Aziende Socio Sanitarie Territoriali (ASST) Spedali Civili” and in a private clinical laboratory in Cagliari. Measles, mumps, rubella and varicella positivity was measured by using commercial IgG Immunoassays, according to the manufacturer’s instruction. Anti-measles IgG, anti-mumps IgG and anti-varicella IgG antibodies were analyzed by chemiluminescence using respectively LIAISON^®^ Measles IgG, Mumps IgG, VZV IgG, (DiaSorin S.p.A., Saluggia (VC), Italy); anti-rubella IgG antibodies were analyzed by chemiluminescence using Access Rubella IgG (Beckman-Coulter, Fullerton, CA, USA), TGS TA Rubella IgG (TECHNOGENETICS S.r.l., Milano (MI), Italy), and LIAISON^®^ Rubella IgG (DiaSorin S.p.A., Saluggia (VC), Italy). Antibodies were defined as qualitative values (positive, negative or equivocal) according to the manufacturer’s guidelines. Equivocal serologic test results were considered negative, as recommended by the Centers for Diseases Control and Prevention [21].

### 2.4. Statistical Analysis

The sample size was determined using previous results of studies conducted on Italian HCW. In particular, we chose 1.96 as the value of standard error, corresponding to a 95% confidence interval (CI); the margin of error was chosen to be equal to 0.05; as an estimate of the population proportion, we used the lowest global seropositivity rate reported in Italian workers occupationally exposed to measles, mumps, rubella and varicella (that is 78%, as described by Campagna et al. for mumps seropositivity) [22,23,24]. The sample size value was assessed to be 264.

Proportions within each category, median ages at the sera samples collection, the seropositivity, as well as its 95% CI were calculated. The Pearson chi square and Fisher-Freeman–Halton’s exact test were computed to compare the proportion of anti-measles, anti-mumps, anti-rubella and anti-varicella IgG titers between groups (considering birthplace, occupation, year of the test and age classes). A multi-variable logistic regression analysis was conducted to evaluate correlation between variable and IgG seroprevalences. A *p* value lower than 0.05 was considered statistically significant (determined using a two-tailed test). The analysis was conducted with SPSS^®^ v20 (IBM Corp, Armonk, NY, USA).

## 3. Results

**Figure 1:**
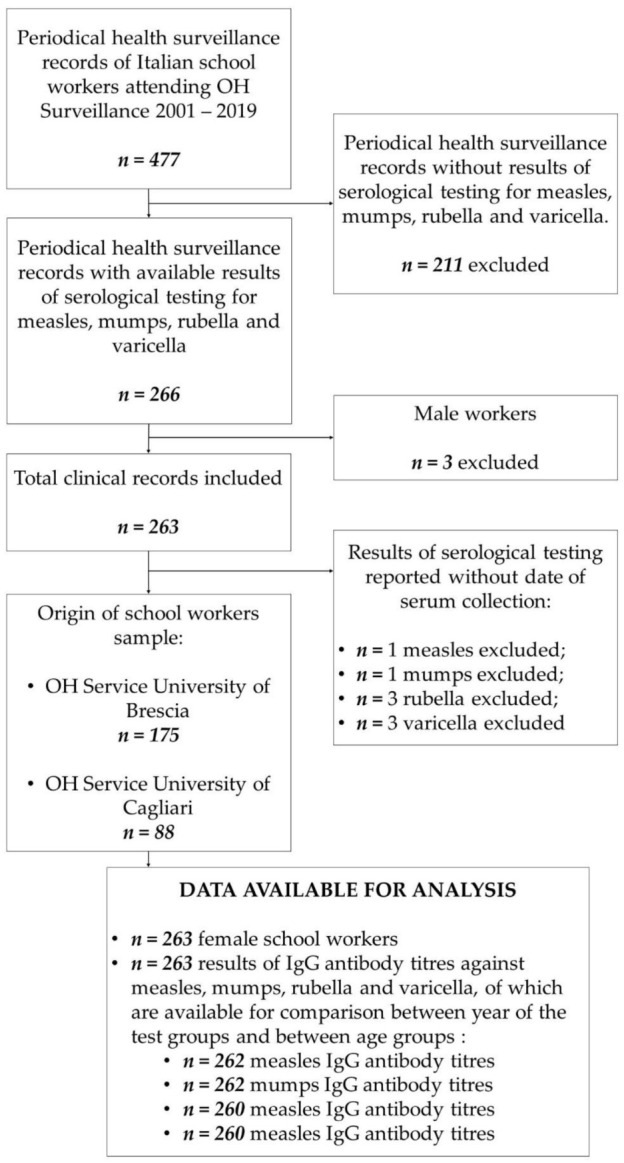
Flow chart of the composition of the study population. Abbreviation: OH, Occupational Health.

In total, 477 periodical health surveillance records of school workers who underwent medical examination during the period 2001–2019 were reviewed. Results of all required serological tests (measles, mumps, rubella and varicella IgG antibody titers) were available for 266 Italian school workers. Among them, only three men (1.1%) respected all inclusion criteria. Due to the small number size, male workers were excluded from the study to preserve cohort homogeneity. Thus, our final cohort accounted for 263 female school workers (98.9%). In total, 175 participants have been included among those admitted to the Occupational Health Service of the University of Brescia and 88 among those admitted to the Occupational Health Service of the University of Cagliari (see Figure 1).

In some cases, the date of sera collection was not reported, due to incomplete recording; for that reason, the comparison between the year of the test groups and between age groups was conducted on 262 (99.6%), 262 (99.6%), 260 (98.9%) and 260 (98.9%) titers (respectively, for measles, mumps, rubella and varicella IgG antibody titers) (see Figure 1).

195 (74.1%) of them came from North Italy, 5 (1.9%) were from Centre Italy, 53 (20.2%) from South Italy and major Islands, 10 (3.8%) were born in Foreign Countries. In total, 117 school workers (44.5%) were teachers in pre-primary schools, 117 (44.5%) were early childhood educators occupied in children’s centers and 29 (11%) had other jobs in both centers. The median age at the moment of measles, mumps, rubella and varicella titers collection was 36.2, 36.2, 35.4 and 36.2, respectively. School workers whose sera were collected before 2015 were more than those tested after 2015 (measles: 145, 55.1%; mumps: 145, 55.1%; rubella: 146, 55.5%; varicella: 144, 54.8%) (Table 1).

### Distribution of the Seropositivity

In Table 2 seropositivities, including exact 95% CI stratified by birthplace, occupation and age at the date of the antibody determination are reported.

The overall seropositivity was 90.5% (CI 95%: 87.0, 94.0) for measles, 85.2% (CI 95%: 80.9, 89.5) for mumps, 94.7% (CI 95%: 92.0, 97.4) for rubella and 97.3% (CI 95%: 95.3, 99.3) for varicella (Table 2).

A significant difference (*p* = 0.001) was found stratifying rubella seropositivity by birthplace, and the highest seropositivity was observed in school workers born in North Italy (97.4%, CI 95%: 95.2, 99.6); rubella seropositivity from Centre Italy, South Italy and Islands and from Foreign Countries were 60.0% (CI 95%: 17.1, 100), 88.7% (CI 95%: 80.2, 97.2) and 90.0% (CI 95%: 71.4, 100), respectively. No statistically significant difference was observed between birthplace groups, in relation to measles, mumps and varicella seropositivity (Table 2).

Considering occupation groups, a statistically significant difference was described between mumps seropositivity (*p* = 0.001), with the highest coverage among teachers (93.2%, CI 95%: 88.6, 97.8), followed by early childhood educators (81.2% CI 95%: 74.1, 88.3), and other occupations (69.0%, CI 95%: 52.2, 85.8). The difference between occupational-group specific seropositivities was not significant in relation to measles, rubella and varicella (Table 2).

Seropositivity of measles, rubella and varicella IgG antibodies was higher in the group of school workers tested before 2015, than in those tested after 2015. More specifically, in the first group rates of positivity were 95.2 (CI 95%: 91.7, 98.7) for measles, 97.9 (CI 95%: 95.6, 100) for rubella and 97.9 (CI 95%: 95.6, 100) for varicella; in those tested after 2015 rates were 84.6 (78.1, 91.1) for measles, 90.4 (CI 95%: 85.0, 95.8) for rubella and 96.6 (CI 95%: 93.3–99.9) for varicella. The difference between before- and after-2015 groups was statistically significant for measles and rubella (*p* = 0.005 and *p* = 0.01, respectively). In relation to mumps, the prevalence of protected individuals was lower in the group tested before 2015, but the difference was not statistically significant (Table 2).

No statistically significant difference was observed comparing age groups for all studied diseases (Table 2).

Concerning male seropositivity, two out of three (66.7%) were anti-measles IgG positive, and all of them were anti-mumps, anti-rubella and anti-varicella IgG positive (data not shown in the Table 2)

Multi-variable logistic regression analysis showed an association between birthplace groups and measles IgG seroprevalence that highlighted a higher seroprevalence among workers from the rest of Italy. In this respect, odds ratio of the Rest of Italy group was 12.7 (Table 3).

Concerning occupation groups, a statistically significant association were observed between other group and mumps IgG seropositivity. In this regard, an odds ratio of 0.13 (0.04–0.48) was observed. Moreover, statistically significant lower seroprevalences of measles and mumps IgG antibodies were observed (Table 3).

Finally, statistically significant associations were detected between after 2015 and ≥40 groups and measles IgG seroprevalences (Table 3).

## 4. Discussion

To our knowledge, this is the first study that evaluated the prevalence of non-immune subjects against measles, mumps, rubella and varicella at scholar services, in particular from early childhood to pre-primary school workers. Results from our study showed an overall seronegativity of 9.5% for measles, 14.8% for mumps, 5.3% for rubella and 2.7% for varicella. As previously observed in other Italian job categories that are occupationally exposed to measles, mumps, rubella and varicella, such as HCWs [22,23,24], a proportion of non-immune school workers, in particular teachers and childhood educators, were observed. This results in a not negligible number of unprotected school workers and highlighted a key need: within the school sector, where the risk of contact with vaccine-preventable diseases is high, it would be desirable to achieve global seropositivity close to 100% to protect both school workers and third parties. Our results showed a specific occupational risk of contracting infectious diseases in school workers, due to susceptible subjects exposed to a working environment where the viruses have a higher spreading compared with the general environment. In a study conducted by Harris et al. before the introduction of routine vaccinations, it was seen an increased risk in mumps acquisition in school workers compared with university staff. In that study, kindergarten teachers acquired as many occupation-associated cases of mumps as reported in the physician group [25]. More recently, the same conclusion was reached by Macintosh et al. that described how schools represent an ideal environment for infectious disease spreading, thanks to the concurrence of multiple factors including densely populated and confined environments and frequent and close contacts between students, teachers and staff [17,26]. Taken into account such considerations, it is crucial to ensure that each school worker, regardless of the specific task, is covered, following the same indications proposed for HCWs [27].

Further considerations can be developed in the light of non-uniform global seropositivity. In fact, in relation to measles and rubella, the found rates could be more associated with the action of vaccination campaigns. In Italy, the measles vaccine has been available since 1976; since 1979 it is recommended to all children aged 15 months until the 1990s [28]. Additionally, the rubella vaccine has been available since 1972. Initially, this latter vaccination was recommended only for prepubescent girls; subsequently, since the 1990s, with the introduction of the trivalent measles-mumps-rubella vaccines (MMR vaccine), it was extended to children of both sexes below the age of 2 years [29]. On the other hand, in relation to mumps, the introduction of vaccination has only taken place since the 1990s, with the introduction of the trivalent vaccine [30]. It is therefore likely that the observed seropositivity is to be associated almost exclusively with previous infections. Likewise, being varicella vaccine universally available to newborns only from 2005 (and only in some regions) [31], it is likely that the prevalence of protected workers is to be associated almost exclusively with previous varicella infections.

In respect of birthplace, up to 40% of Centre Italy school workers were not immune to rubella and the difference between seropositivities was statistically significant (*p* = 0.001). However, based on the results of the logistic regression analysis, North Italy showed significantly lower measles seroprevalences, in comparison with the rest of Italy. These results could be in part justified by different vaccination policies along with the Italian territory. In fact, notwithstanding national recommendations, implementation of a non-compulsory vaccine plan is under the responsibility of each Italian region [32]. For this reason, Italy presents a mosaic of different regional vaccination campaigns. These campaigns include different methods of intervention (incentives to physicians, information programs aimed at the general population, specific training for operators) [33]. It is likely that over the years these different regional vaccine plans have influenced the creation of heterogeneous seropositivity along with the Italian territory.

Concerning the prevalence of mumps immune school workers, comparing job groups, a statistically significant difference was observed (*p* = 0.001) and other jobs showed the lowest rates (69.0%) (Table 2). Results of the logistic regression also pointed out a statistically significant increased risk of seronegativity in this group, with an odds ratio of 0.13 (95% CI 0.04–0.48, *p* = 002). This latter group mostly consisted of janitors and cafeteria workers. Lower educational attainment may have contributed to refusing vaccination [34], and thus it may be associated with the observed low prevalence of mumps immune workers. As a matter of fact, previous studies explored determinants of vaccine hesitancy. In a systematic review in Latin America, Guzman-Holst et al. reported that the greatest concerns leading to vaccine refusal were observed in individuals with a low educational level [35]. Nevertheless, it is noteworthy that a preceding systematic review carried out by Larson et al. described the level of education as having different effects, acting both as a promoter and as a potential barrier to vaccination in light of different cultural and social contexts [36]. However, the low rate of mumps-immune school workers in the early childhood educator group (81.2%) is remarkable. This lower seroprevalence of mumps antibodies in early childhood educators was also observed computed logistic regression analysis. The same analysis also highlighted a lower measles seroprevalence in the early childhood educators. Such a prevalence may lead to two main consequences. First, this category is the one exposed to the youngest pupils (aged from 3 months to 3 years old). Harris et al. observed that the rate of occupation acquired mumps cases among teachers showed an inverse proportion with the age of their students [25]. At the same time, the attack rate in children increases inversely with age. In fact, Barrabeig et al. reported that during a measles outbreak in educational centers, the highest attack rate was observed among children aged 6 to 11 months, followed by those aged between 12 and 23 months. The author attributes the absence of immunity in these age groups to the lack of contact with the virus, to having not received vaccination yet and to the loss of maternal immunity [37]. Therefore, early childhood educators could be potentially exposed to a higher risk than teachers. On the other hand, by having contact with children aged from 3 months to 3 years, early childhood educators could become themselves carriers of pathologies and infect those who have not yet been vaccinated, as seen in HCWs to vulnerable patients [18], and in a case report in Italy where a previously unvaccinated teacher who acquired measles infection during a tour abroad, once returned home, introduced the measles virus into Pordenone area (North Italy), causing an outbreak that involved eight adolescents and young adults [38]. In relation to measles and rubella, the category of early childhood educators shows the lowest immunity rates between occupation groups, but in this case, the difference was not statistically significant.

The results of the present study pointed out a significant difference between seropositivity for measles and rubella in the group tested before and in that tested after 2015. More specifically, the group of school workers tested after 2015 showed rates of positivity for measles and rubella of 10.6% (from 95.2% to 84.6%, *p* = 0.005) and 7.5% (from 97.9% to 90.4%, *p* = 0.01), respectively, lower than the group tested before 2015. These decreases in the seroprevalence of IgG antibodies were also observed concerning measles on the logistic regression. In fact, workers tested after 2015 had a higher risk of mumps IgG antibody seronegativity, with a 0.17 odds ratio (95% CI 0.06–0.48, *p*= 0.001). These results contrast with what was expected after 2015. In fact, WHO previously set 2015 as a target year for the elimination of measles and rubella in the WHO European Region [20]. A similar reduction was also observed in Italian childhood vaccine coverage [6]. The decrease of seropositivity rates observed after 2015 may be due to the misinformation and the vaccine hesitancy diffusion, at least partially. This phenomenon interested several countries and Italy was not spared. It led to significative effects on immunization choices, determining a drop in vaccine immunization rates and resulting in an increase of vaccine-preventable diseases outbreaks [39]. In this regard, first Luthy et al. [16], and later Macintosh et al. [17,26], explored vaccine perceptions among US school employees. Critical gaps in vaccination knowledge were observed. In particular, many school workers were unsure of their vaccination status [16], and even though many of them thought vaccinations were important for school-aged children, a lower percentage believed vaccinations were important for adults [16,17]. Moreover, about the reason for not receiving a second MMR vaccination or an MMR vaccination as an adult, the most common response was “Was not sure I needed one” [16,17,26]. Similar results were also reported by Riccò et al. in North Italy teachers. In fact, only 5.9% of the interviewed teachers were able to correctly recall the recommendations of the Italian National Immunization Prevention Plan. The same study also showed that only 22.6% of participants knew that measles is a potentially severe disease [40].

As far as the age group is concerned, a significantly higher seroprevalence of anti-measles IgG antibodies was observed in the older group, based on the results of the logistic regression. This is consistent with previous studies, that highlighted the highest antibody seropositivity in older people (usually over 50) [19,41,42,43]. We can speculate that the highest proportion of measles serologically immune subjects in the age class ≥40 may be the consequence of a natural infection exposition and not secondary to vaccination. As matter of fact, it is known that immunity secondary to natural disease is associated with higher IgG antibody titers, and it is likely to be maintained for life [44,45,46]. Furthermore, using the variable age as a proxy of the length of service and consequently of longer exposure to biological occupational risk, it can be hypothesized that over the years older groups have had a greater probability of coming into contact with the natural disease and that this has acted as a natural booster.

The results of our study pointed out a low proportion of men employed in the school sector. As a matter of fact, after excluding workers based on the inclusion criteria, our cohort consisted of only three men. This result was in line with what the Organisation for Economic Cooperation and Development (OECD) reported: Italy is one of the Countries with the highest number of female teachers: in 2016, 99% of pre-primary school teachers and 96% of primary school teachers were female [47]. Moreover, in the same year the OECD reported that the rate of teachers under 50 in primary schools was 45% [47]. It is known that these diseases can cause severe complications in pregnant women, including premature birth, teratogenic effects and abortion [48,49,50,51], so that these potential severe adverse health effects should also be considered in the biological risk assessment of female school workers.

Possible initiatives aimed at reducing vaccine hesitancy, especially within school workers could be represented by the execution of systematic and widespread health surveillance, which includes useful screening strategies, proper risk communication and effective vaccination policies. Screening strategies conducted by means of serological testing due to recall history have a low negative and positive predictive value [52], and are, therefore, insufficient in order to assess the coverage against diseases preventable with vaccinations [22]. The evaluation of the antibody titer during employment and any subsequent vaccination could be strongly recommended for all workers in the school sector, due to the low costs, especially considering the social expenses linked to potential outbreaks [53]. In susceptible women, a temporary alternative duty during the period of pregnancy should be prescribed in order to avoid exposure.

This study had some limitations: data were derived from a small sample of 263 female school workers, attending occupational health surveillance in two Italian Occupational Health Services. Nevertheless, the size of our sample is consistent with the value of 264 participants estimated through sample size determination. Additionally, it should be considered that Italian school teachers are represented mainly by women: in 2017 female teachers accounted for 99% and 96% in pre-primary and primary institutions, respectively [54]. For those reasons, it could be supposed that our results reflect the characteristics of the Italian school worker population. We did not estimate if the lack of vaccination immunity was either linked to the waning of antibody coverage over the years or was linked to an ineffective vaccination. Furthermore, no type of vaccine received by workers was described. In fact, only a small proportion of the examined clinical records clearly reported documented receipt of virus vaccine with attached vaccine records; most of the examined clinical records only contained a self-reported history of disease and/or vaccination, which did not accurately predict immunity, as stated by Trevisan et al. and Campagna et al. [22,52], and we decided not to collect them.

Despite some limitations, this work has numerous strengths. Studies on school environment are few and mostly limited to describing outbreak cases or investigating the determinants of hesitancy among teachers and childcare workers. Several previous studies showed the effective presence of an occupational biological risk in the school sector. Nevertheless, as of today, this work represents the first and only study investigating measles, mumps, rubella and varicella seroprevalence in the school worker population. Our study was not limited to reporting global seroprevalence data, but it describes the results of the stratification of the analyses for homogeneous groups, highlighting the ones most at risk within the studied cohort. For the occupational physician these works are extremely important to identify which workers are the most susceptible to the vaccine-preventable diseases and thus to assess the specific occupational risk. In the light of these various assessments, it is possible to plan specific strategies of protection. These include: information addressed to the individual worker during the occupational visit, courses on biological risk addressed to homogeneous groups, up to the inclusion of modification on the assessment of fitness for work. Furthermore, it constitutes a fundamental tool for policymakers who can carry out either political decisions or public health strategies. Finally, this study confirms the importance of health surveillance and immunological screening in school settings and more generally in all work environments with specific occupational risks.

## 5. Conclusions

Even considering the outbreaks and the re-emerging of measles, mumps, rubella and varicella, it is still missing widespread monitoring of worker’s immunity within school environments. This lack of data, together with a general underestimation of the risk associated with these diseases, may delay the process of eradication of vaccine-preventable diseases and can expose school workers and third parties, such as students and families, to avoidable risks.

## Figures and Tables

**Table 1:** Characteristics of the study population (N = 263 female school workers), Italy. 2001–2019.

Variable				N		(%)		Median Age at the Time of Antibody Determination (IQR)				
**Birthplace**												
North Italy				195		(74.1)						
Centre Italy				5		(1.9)						
South Italy and Islands				53		(20.2)						
Foreign Country				10		(3.8)						
**Occupation**												
Teacher				117		(44.5)						
Early childhood educator				117		(44.5)						
Other				29		(11)						
**Disease**												
Measles								36.2			(30.0–42.7)	
Mumps								36.2			(29.8–42.7)	
Rubella								35.4			(29.7–42.3)	
Varicella								36.2			(29.9–43.0)	
	**Measles**		**Mumps**				**Rubella**			**Varicella**		
	N	(%)	N		(%)		N		(%)	N		(%)
**Year**												
Before 2015	145	(55.1)	145		(55.1)		146		(55.5)	144		(54.8)
After 2015	117	(44.5)	117		(44.5)		114		(43.4)	116		(44.1)
**Age**												
<30	63	(24.0)	66		(25.1)		66		(25.1)	64		(24.3)
30–39	109	(41.4)	106		(40.3)		108		(41.1)	106		(40.3)
40–49	65	(24.7)	66		(25.1)		63		(24.0)	64		(24.3)
≥50	25	(9.5)	24		(9.1)		23		(8.7)	26		(9.9)

Abbreviation: IQR, Interquartile Range.

**Table 2:** Distribution of the seropositivity of measles, mumps, rubella and varicella.

Variables	Seropositivity							
	Measles		Mumps		Rubella		Varicella	
	%	(95% CI)	%	(95% CI)	%	(95% CI)	%	(95% CI)
**Total sample**	90.5	(87.0–94.0)	85.2	(80.9–89.5)	94.7	(92.0–97.4)	97.3	(95.3–99.3)
**Birthplace**								
North Italy	88.2	(83.7–92.7)	84.6	(79.5–89.7)	97.4	(95.2–99.6)	96.9	(94.1–99.1)
Centre Italy	100	(100)	100	(100)	60.0	(17.1–100)	80.0	(44.9–100)
South Italy and Islands	98.1	(94.4–100)	86.8	(77.7–95.9)	88.7	(80.2–97.2)	100	(100)
Foreign Country	90.0	(71.4–100)	80.0	(55.2–100)	90.0	(71.4–100)	100	(100)
*p* value	0.11		0.84		0.001		0.11	
**Occupation**								
Teacher	94.0	(89.7–98.3)	93.2	(88.6–97.8)	95.7	(92.0–99.4)	96.6	(93.3–99.9)
Early childhood educator	85.5	(79.1–91.9)	81.2	(74.1–88.3)	92.3	(87.5–97.1)	98.3	(96.0–100)
Other occupation	96.6	(90.0–100)	69.0	(52.2–85.8)	100	(100)	96.6	(90.0–100)
*p* value	0.55		0.001		0.27		0.63	
**Year of test**								
Before 2015	95.2	(91.7–98.7)	82.8	(76.7–88.9)	97.9	(95.6–100)	97.9	(95.6–100)
After 2015	84.6	(78.1–91.1)	88.0	(82.1–93.9)	90.4	(85.0–95.8)	96.6	(93.3–99.9)
*p* value	0.005		0.30		0.01		0.70	
**Age** (**years**)								
<30	87.3	(79.1–95.5)	93.9	(88.1–99.7)	93.3	(87.3–99.3)	98.4	(95.3–100)
30–39	88.1	(82.0–94.2)	79.2	(71.5–86.9)	95.4	(91.4–99.4)	94.3	(89.9–98.7)
40–49	93.8	(87.9–99.7)	86.4	(78.1–94.8)	92.1	(85.4–98.8)	100	(100)
≥50	100	(100)	83.3	(68.4–98.2)	100	(100)	100	(100)
*p* value	0.18		0.07		0.56		0.14	

**Table 3:** Results of multi-variable logistic regression analysis. Relationship between workers’ characteristics and measles, mumps, rubella and varicella seropositivity was assessed.

Variables	Measles			Mumps			Rubella			Varicella		
	Odds Ratio (95% CI)		*p* Value	Odds Ratio (95% CI)		*p* Value	Odds Ratio (95% CI)		*p* Value	Odds Ratio (95% CI)		*p* Value
**Birthplace**												
North Italy (Reference)	1.00			1.00			1.00			1.00		
Rest of Italy	12.7	(1.6–101.3)	0.02	1.27	(0.50—3.22)	0.62	0.20	(0.06–0.69)	0.20	1.76	(0.19–16.2)	0.62
**Occupation**												
Teacher (Reference)	1.00			1.00			1.00			1.00		
Early childhood educator	0.31	(0.11–0.86)	0.02	0.34	(0.14–0.80)	0.01	0.38	(0.11–1.36)	0.14	2.47	(0.44–14.07)	0.31
Other	0.24	(0.02–2.72)	0.25	0.13	(0.04–0.48)	0.002	N.C. *	N.C. *	N.C. *	0.10	(0.01–1.60)	0.10
**Year of test**												
Before 2015 (Reference)	1.00			1.00			1.00			1.00		
After 2015	0.17	(0.06–0.48)	0.001	0.99	(0.44–2.23)	0.98	0.38	(0.95–1.54)	0.18	0.32	(0.06–1.83)	0.20
**Age** (**years**)												
<40 (Reference)	1.00			1.00			1.00			1.00		
≥40	4.22	(1.11–16.04)	0.03	1.25	(0.52–3.01)	0.62	1.02	(0.28–3.76)	0.97	N.C. *	N.C. *	N.C. *

* N.C.: Not calculated. These odds ratios were not computed because workers within each group had 100% seroprevalence.

## Data Availability

The data presented in this study are available on request from the corresponding author.
